# Relationship between circulating inflammatory factors and glioma risk and prognosis: A meta‐analysis

**DOI:** 10.1002/cam4.2585

**Published:** 2019-10-09

**Authors:** Yuan Feng, Jia Wang, Dezhong Tan, Peng Cheng, Anhua Wu

**Affiliations:** ^1^ Department of Neurosurgery The First Hospital of China Medical University Shenyang Liaoning People's Republic of China; ^2^ Department of Neurosurgery The First Affiliated Hospital of Xi'an Jiaotong University Xi'an Shanxi People's Republic of China; ^3^ Center of Brain Science The First Affiliated Hospital of Xi'an Jiaotong University Xi'an Shanxi People's Republic of China; ^4^ Department of Otorhinolaryngology Head and Neck Surgery The First Hospital of China Medical University Shenyang Liaoning People's Republic of China

**Keywords:** circulating inflammatory factors, glioma, prognosis, risk

## Abstract

**Background:**

Inflammatory factors have been considered a significant factor contributing to the development and progression of glioma. However, the relationship between circulating inflammatory factors and glioma risk as well as their prognostic values in glioma patients is still inconclusive. Here, we performed a meta‐analysis to address this issue.

**Methods:**

Relevant articles were identified through PubMed, EMBASE, the Cochrane Library, Web of Science, Wanfang database, and China National Knowledge Infrastructure (CNKI) from inception to February 2019. The weighted mean differences (WMDs) or standard mean differences (SMDs) with 95% confidence intervals (CIs) were used to describe the predictive ability of the levels of circulating inflammatory factors on glioma risk. To evaluate the prognostic values of the circulating inflammatory factors in glioma, hazard ratios (HRs) with 95% CIs were used.

**Results:**

Thirty‐one studies comprising 2587 patients were included. The overall analysis showed that increased circulating interleukin‐6 (IL‐6) [SMD 0.81 (95% CI: 0.21‐1.40; *P* = .008)], interleukin‐8 (IL‐8) [SMD 1.01 (95% CI: 0.17‐1.84; *P* = .018)], interleukin‐17 (IL‐17) [SMD 1.12 (95% CI: 0.26‐1.98; *P* = .011)], tumor necrosis factor‐*α* (TNF‐*α*) [SMD 1.80 (95% CI: 1.03‐2.56; *P* = .000)], transforming growth factor‐*β* (TGF‐*β*) [SMD 10.55 (95% CI: 5.59‐15.51; *P* = .000)], and C‐reactive protein (CRP) [SMD 0.95 (95% CI: 0.75‐1.15; *P* = .000)] levels were significantly associated with glioma risk. On the other hand, our results showed that circulating IL‐6 [HR 1.10 (95% CI: 1.05‐1.16; *P* = .000)] and CRP [HR 2.02 (95% CI: 1.52‐2.68; *P* = .000)] levels were highly correlated with a poor overall survival (OS) rate in glioma patients.

**Conclusion:**

Our results indicate that increased circulating IL‐6, IL‐8, IL‐17, TNF‐*α*, TGF‐*β*, and CRP levels are significantly associated with increased glioma risk. Moreover, our meta‐analysis suggests that circulating IL‐6 and CRP may serve as powerful biomarkers for a poor prognosis in glioma patients.

## Graphical abstract

Inflammatory factors have been considered a significant factor contributing to the development and progression of glioma. Our meta‐analysis indicates that increased circulating IL‐6, IL‐8, IL‐17, TNF‐*α*, TGF‐*β*, and CRP levels are significantly associated with increased glioma risk. Moreover, it suggests that circulating IL‐6 and CRP may serve as powerful biomarkers for a poor prognosis in glioma patients.

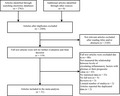

## 1 INTRODUCTION

Primary brain cancer is composed of tumors that originate from within the central nervous system (CNS) and comprises a large number of different kinds of tumors with a benign to malignant status.^1^ Gliomas represent approximately 75% of malignant brain tumors in adults^2^ and are the most common primary tumor of the CNS. Patients receiving treatment, including aggressive surgery followed by chemotherapy and radiotherapy, still experience an extremely poor clinical outcome due to the highly proliferative and aggressive nature of this tumor.^3^, ^4^ Considering the poor clinical outcome after standard treatment, a set of biomarkers or a whole sample profile for the early detection of glioma is currently under intensive investigation.^5^ At the same time, using several inflammatory markers to predict patient prognosis has also proven to be a viable strategy.^6^ Without a doubt, the identification of these indicators of glioma could provide a variety of advantages, such as the early intervention of treatment, a reduction in morbidity and mortality, and monitoring the progression of treatment. Currently, there are no specific serological biomarkers for gliomas, which suggests that the development of an assessment for the association between circulating indicators and the risk of glioma as well as their prognostic values in glioma is extremely indispensable.

Inflammatory factors have been considered a significant factor contributing to the complexity and lethality of glioblastoma multiforme (GBM), which can evade immune surveillance by creating an immunosuppressive tumor microenvironment.^7^ The expression of a few genes in connection with inflammation was previously demonstrated as an important contributing factor in glioma development, including tumor promotion and progression.^8^ Therefore, as essential mediators linking inflammation and cancers, inflammatory factors are attracting a large amount interest of researchers who are searching potential therapeutic and prognostic biomarkers for cancers. Previous studies have shown that several circulating markers of inflammation, such as interleukin‐6 (IL‐6), tumor necrosis factor‐*α* (TNF‐*α*), and C‐reactive protein (CRP), were observably higher in patients with glioblastoma compared to healthy controls.^9^ Accumulating evidence has also shown that these markers may be involved in the risk and survival of glioma. However, the association between circulating inflammatory factors and glioma risk or prognosis remains controversial. Some research has suggested that CRP and IL‐6 could effectively predict clinical outcomes in patients with glioma.^6^, ^10^, ^11^, ^12^, ^13^ In contrast, other studies showed no association between these factors and the prognosis of patients with glioma.^10^, ^14^, ^15^, ^16^ To elucidate the essential relationship between circulating inflammatory factors and the risk of glioma as well as their prognostic values in glioma in clinical practice, we performed this meta‐analysis.

## 2 MATERIALS AND METHODS

### 2.1 Retrieval strategy

Potentially relevant articles were searched using electronic databases and manual retrieval. We systematically retrieved PubMed, EMBASE, the Cochrane Library, Web of Science, Wanfang database, and China National Knowledge Infrastructure (CNKI) for articles published up to February 2019. There were no restrictions of languages or sources through an initial search of published articles. The following retrieval terms were used: “cytokines,” “interleukins,” “C‐reactive protein (CRP),” “tumor necrosis factor (TNF),” “transforming growth factor beta (TGF‐*β*),” “blood,” “serum,” “plasma” and “glioma.” We manually searched the reference lists of related major studies and reviews for additional citations. Moreover, the references of the retrieved articles were screened for other possibly eligible reports.

### 2.2 Selection criteria

Eligible studies had to meet the following criteria: (a) the patients in the studies were diagnosed with glioma by pathologists; (b) the studies aimed to explore the different levels of circulating inflammatory factors between glioma patients and controls or the prognostic value of circulating inflammatory factors in glioma; (c) for studies analyzing the relationship between preoperative circulating inflammatory indicators and the risk of glioma, the mean value and standard deviation (SD) of the circulating inflammatory factors' levels for glioma patients and controls were provided directly or could be converted from the materials given in the articles; for prognostic studies, the hazard ratio (HR) and 95% confidence interval (CI) of the circulating inflammatory factors' levels for overall survival (OS) were supplied directly or could be converted from survival data or the Kaplan‐Meier curve given in the articles using the methods of Tierney et al^17^; and (d) for duplicate data reported by the same author, the most complete and recently published study was reviewed.^18^

The exclusion criteria were as follows: (a) studies measured the levels of circulating inflammatory factors after treatment or measured tissue cytokines; (b) comments, reviews, clinical guidelines, letters to the editor, or case reports; and (c) incomplete data in the original studies made it impossible to obtain the mean value, SD, HR, and its 95% CI. Due to the lack of relevant articles (less than two), some inflammatory factors, such as interleukin‐2 (IL‐2), interleukin‐5 (IL‐5), interleukin‐7 (IL‐7), interleukin‐16 (IL‐16), and interleukin‐18 (IL‐18), were also excluded from the meta‐analysis.

### 2.3 Data extraction

All information and data were extracted from each relevant article by two investigators independently. Any further inconsistencies were addressed by a joint discussion. The following study characteristics were recorded from each eligible study: name of the first author, publication year, country, number of patients and controls, study participants' age, gender, sample type, WHO grade, and inflammatory factors assay method. Furthermore, we extracted the cut‐off point, maximum follow‐up time, and type of HR from each survival study. For studies analyzing the relationship between inflammatory indicators and the risk of glioma, we extracted the outcome information, including the mean value and SD of circulating inflammatory factors' levels for glioma patients and controls. For prognostic studies, the HR estimated with its 95% CI for OS was extracted. When the mean value and SD were not supplied directly in the study, we contacted the authors for unpublished data or extracted them using the methods of Hozo et al^19^ and Wan et al^20^ If HRs and their 95% CIs were not supplied directly in the studies, we also contacted the authors for additional data or calculated them from the survival curves using the methods of Tierney et al^17^

### 2.4 Quality assessment and statistical analysis

The methodological quality of the eligible studies was assessed using the Newcastle‐Ottawa Scale (NOS),^21^ which concentrated on patient selection, comparability, and exposure factor/outcomes. The total NOS scores ranged from 0 to 9, and a score ≥ 6 was considered high quality.^22^ The weighted mean differences (WMDs) or standard mean differences (SMDs) with 95% CIs were used to describe the predictive ability of the circulating inflammatory factors' levels on glioma risk. To evaluate the prognostic value of circulating inflammatory factors in glioma, HRs with 95% CIs were used in the meta‐analysis. Heterogeneity was estimated by Cochrane's Q test and *I* ^2^ measurement among the studies, and multiple individual studies were considered to have moderate or high heterogeneity when *P* *≤* .10 or *I* ^2^ ≥ 50%. WMDs, SMDs, and HRs were pooled using a random effects model if heterogeneity was significant (*I* ^2^ *≥* 50%). Otherwise, we used the fixed effects model. Subgroup analyses were used to explore the potential sources of heterogeneity. A sensitivity analysis was performed to evaluate the availability and reliability of the results. Funnel plots with Begg's test and Egger's test were used to research publication bias.^23^ The effects of circulating inflammatory factors on the risk and survival of glioma patients were considered statistically significant if the two‐tailed *P*‐value was < .05. The quantitative meta‐analysis was conducted using STATA version 12.0 (StataCorp LP).

## 3 RESULTS

### 3.1 Characteristics and quality evaluation of the included studies

**Figure 1:**
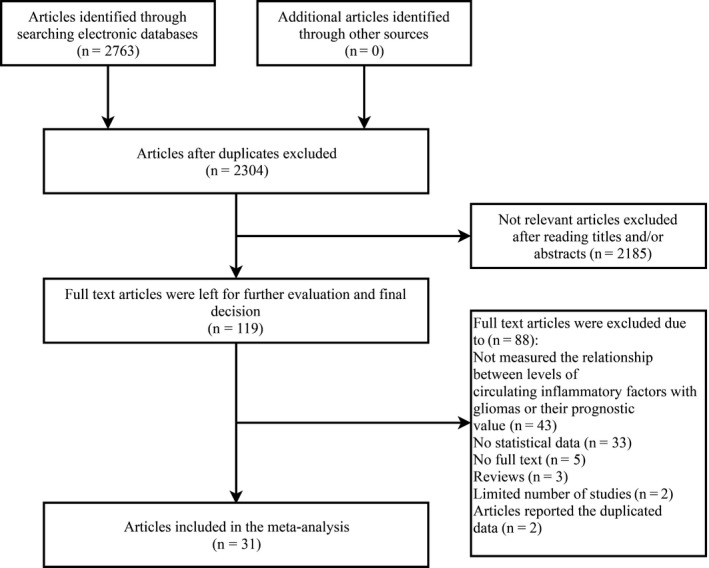
Flowchart of studies selection process for meta‐analysis

The detailed retrieval processes of relevant articles are demonstrated in Figure 1. Based on the selection criteria, a total of 2763 articles were identified by electronic databases and manual retrieval in the primary search. After excluding duplicate articles, there were 2304 remaining studies. Next, 2185 irrelevant articles were excluded after reading the titles and/or abstracts. A total of 119 full‐text articles remained for further evaluation and a final decision, and 88 articles were excluded due to the following reasons: 43 did not assess the relationship between the levels of circulating inflammatory factors with gliomas or their prognostic values in glioma, 33 had no statistical data, five were not full‐text articles, three were reviews, two had a limited number of studies (These two articles studied the relationship between IL‐7 and IL‐18 and gliomas. But there were fewer than two studies about IL‐7 or IL‐18, the meta‐analysis could not be performed), and two were articles reporting duplicate data. Finally, 31 studies that met the requirements were included in the final meta‐analysis.

Of these 31 studies, 24 articles^9^, ^10^, ^24^, ^25^, ^26^, ^27^, ^28^, ^29^, ^30^, ^31^, ^32^, ^33^, ^34^, ^35^, ^36^, ^37^, ^38^, ^39^, ^40^, ^41^, ^42^, ^43^, ^44^, ^45^ assessed the relationship between blood‐based inflammatory cytokines and glioma risk. Table 1 presents the characteristics and quality evaluation of these 24 included studies. The publication dates ranged from 2000 to 2018. A total of 1950 glioma patients and 1160 controls were included in these studies. Of these 24 studies, 10 examined IL‐6,^9^, ^10^, ^24^, ^29^, ^33^, ^36^, ^37^, ^38^, ^39^, ^43^ five examined IL‐8,^28^, ^30^, ^36^, ^37^, ^44^ four examined interleukin‐17 (IL‐17),^30^, ^33^, ^39^, ^40^ nine examined TNF‐*α*,^9^, ^24^, ^27^, ^29^, ^33^, ^36^, ^37^, ^41^, ^45^ three examined transforming growth factor‐*β* (TGF‐*β*),^25^, ^26^, ^39^ two examined CRP,^9^, ^34^ three examined interleukin‐4 (IL‐4),^33^, ^35^, ^37^ five examined IL‐10,^31^, ^33^, ^36^, ^37^, ^42^ three examined interleukin‐12 (IL‐12),^33^, ^37^, ^44^ two examined interleukin‐23 (IL‐23),^39^, ^40^ and four examined monocyte chemotactic protein‐1 (MCP‐1).^24^, ^28^, ^29^, ^32^ Among these 24 studies, serum samples from patients with glioma were collected in 22,^9^, ^10^, ^24^, ^25^, ^26^, ^27^, ^28^, ^29^, ^30^, ^32^, ^33^, ^34^, ^35^, ^36^, ^37^, ^38^, ^39^, ^40^, ^41^, ^42^, ^43^, ^45^ and plasma samples were collected in the other two.^31^, ^44^ Most of these studies used an enzyme‐linked immunosorbent assay (ELISA) to detect the concentrations of inflammatory cytokines, and only a few studies used other methods, such as bead array technology,^33^ fluorescence microsphere detection,^36^ xMAP,^37^ and radioimmunoassays.^41^, ^45^ For the quality score, all 24 studies scored at least 6 based on NOS scoring and are shown in Table 1.

**Table 1:** The general characteristics of the 24 articles which reported the association between circulating inflammatory cytokines and the glioma risk

Author	Year	Country	Glioma/Control	Age (Glioma/Control)	Male/Female	Sample type	Grade（Ⅰ+Ⅱ/Ⅲ+Ⅳ）	Method	Inflammatory factors	Study quality
Ji^24^	2018	China	104/30	42.21/43.0	76/58	Serum	53/51	ELISA	MCP‐1, IL‐6, TNF‐*α*	7
Zhang^25^	2018	China	70/60	67.3/66.9	69/61	Serum	0/70	ELISA	TGF‐*β*	6
Li^26^	2018	China	188/50	45.9/46.5	121/117	Serum	0/188	ELISA	TGF‐*β*	7
Liu^27^	2017	China	90/52	52.26/53.15	76/66	Serum	43/47	ELISA	TNF‐*α*	7
OMKoper^28^	2017	Poland	20/20	56/56	15/25	Serum	3/17	ELISA	IL‐8, MCP‐1	7
Zhou^29^	2016	China	93/40	45.91/45.85	81/52	Serum	47/46	ELISA	MCP‐1, IL‐6, TNF‐*α*	7
Yang^30^	2016	China	37/37	38.9/38.8	45/29	Serum	18/19	ELISA	IL‐8, IL‐17	6
Mostafa^31^	2016	Germany	51/36	56.8/36.1	NA	Plasma	0/51	ELISA	IL‐10	6
Moogooei^32^	2015	Iran	123/189	53/48	175/137	Serum	0/123	ELISA	MCP‐1	6
Nijaguna^33^	2015	India	148/26	NA	NA	Serum	0/148	Bead array technology	IL‐4, IL‐6, IL‐10, IL‐12, IL‐17, TNF‐*α*	6
Li^34^	2014	China	241/116	49.6/48.3	210/147	Serum	103/138	ELISA	CRP	8
Shamran^35^	2014	Iraq	100/40	40.2/33.9	72/68	Serum	NA	ELISA	IL‐4	7
Li^36^	2013	China	135/61	44.5/NA	NA	Serum	53/82	Fluorescence microsphere detection	IL‐6, IL‐8, IL‐10, TNF‐*α*	6
Albulescu^37^	2013	Romania	55/20	60/57	40/35	Serum	0/55	xMAP	IL‐4, IL‐6, IL‐8, IL‐10, IL‐12, TNF‐*α*	6
Doroudchi^38^	2013	Iran	38/26	34.7/48.6	29/35	Serum	20/18	ELISA	IL‐6	6
Zhu^10^	2011	China	182/49	51.5/41.5	165/66	Serum	77/105	ELISA	IL‐6	7
Gaspar^9^	2011	Spain	40/60	61/65	NA	Serum	0/40	ELISA	IL‐6, TNF‐*α*, CRP	7
Hu^39^	2011	China	35/20	39.3/40.3	32/23	Serum	16/19	ELISA	IL‐17, IL‐23, IL‐6, TGF‐*β*	6
Zhou^40^	2010	China	23/17	NA	NA	Serum	0/23	ELISA	IL‐17, IL‐23	6
Zhou^41^	2010	China	42/55	45.6/49.5	62/35	Serum	NA	Radioimmunoassay	TNF‐*α*	6
Krzyszkowski^42^	2008	Poland	16/28	46.3/49	21/23	Serum	0/16	ELISA	IL‐10	6
Zhang^43^	2004	China	60/65	51/NA	NA	Serum	NA	ELISA	IL‐6	6
Salmaggi^44^	2003	Italy	25/23	NA	NA	Plasma	0/25	ELISA	IL‐8, IL‐12	6
He^45^	2000	China	34/40	NA	42/32	Serum	NA	Radioimmunoassay	TNF‐*α*	6

Abbreviations: ELISA, enzyme‐linked immunosorbent assay; NA, not available; xMAP, xMAP assay was performed according to the manufacturers' protocols, and the plates were analyzed using Luminex 200 system.

On the other hand, only eight studies concentrating on the prognostic significance of circulating inflammatory factors in glioma patients were included. The prognostic values of IL‐6 and CRP in glioma were examined in five^6^, ^10^, ^11^, ^14^, ^15^ and four studies,^10^, ^12^, ^13^, ^16^ respectively. The characteristics and quality evaluation of these prognostic‐related studies are presented in Table 2. Of these eight articles, one study reported the prognostic value of not only IL‐6 but also CRP. In these included studies, all outcome observations were based on OS; four provided the original HR, and four reported the Kaplan‐Meier curves. The publication dates ranged from 2011 to 2018, and the maximum follow‐up time ranged from 28 to 154 months. For IL‐6, five studies with 535 glioma patients were enrolled in the prognostic analysis. Regarding CRP, four studies including 466 glioma patients were selected for the prognostic analysis. On NOS scoring, all eight studies scored at least 7.

**Table 2:** The general characteristics of the articles which reported the prognostic significance of circulating inflammatory factors in glioma

	Author	Year	Country	No. of patients	Age(Years)	Male/Female	Sample type	Cut‐off point	Tumor type	Method	Maximum follow‐up time	Survival analysis	HR	Study quality
IL‐6	Liu^14^	2018	Sweden	205	16‐80	137/68	Serum	NA	Glioma	ELISA	1200 d	OS	Curve	8
	Bunevicius^6^	2018	Lithuania	48	Median 57	NA	Serum	2 pg/mL	HGG	Radioimmunoassay	67 mo	OS	Original	8
	Shan^11^	2015	China	86	NA	37/49	Serum	20 ng/mL	Glioma	ELISA	60 mo	OS	Curve	7
	Chiorean^15^	2014	Romania	14	NA	NA	Serum	42 ng/mL	GBM	ELISA	28 mo	OS	Original	7
	Zhu^10^	2011	China	182	Mean 51.5	134/48	Serum	6.06 pg/mL	Glioma	ELISA	66 mo	OS	Original	7
CRP	Miyauchi^16^	2018	Japan	14	Median 62.5	11/3	Plasma	47.6 fmol/μL	GBM	SWATH‐MS	44 mo	OS	Curve	7
	Nijaguna^12^	2015	India	105	Mean 46.3	75/30	Serum	7.3 μg/mL	GBM	ELISA	47 mo	OS	Original	7
	Strojnik^13^	2014	Slovenia	165	Median 56	105/60	Serum	5 mg/L	Glioma	Turbidimetry	154 mo	OS	Curve	8
	Zhu^10^	2011	China	182	Mean 51.5	134/48	Serum	0.2 mg/dL	Glioma	NA	66 mo	OS	Original	7

Abbreviations: ELISA, enzyme‐linked immunosorbent assay; GBM, glioblastoma multiforme; HGG, high‐grade glioma; NA, not available; No. of patients, number of patients; OS, overall survival; SWATH‐MS, SWATH mass spectrometry analysis.

### 3.2 Circulating inflammatory factors and glioma risk

**Figure 2:**
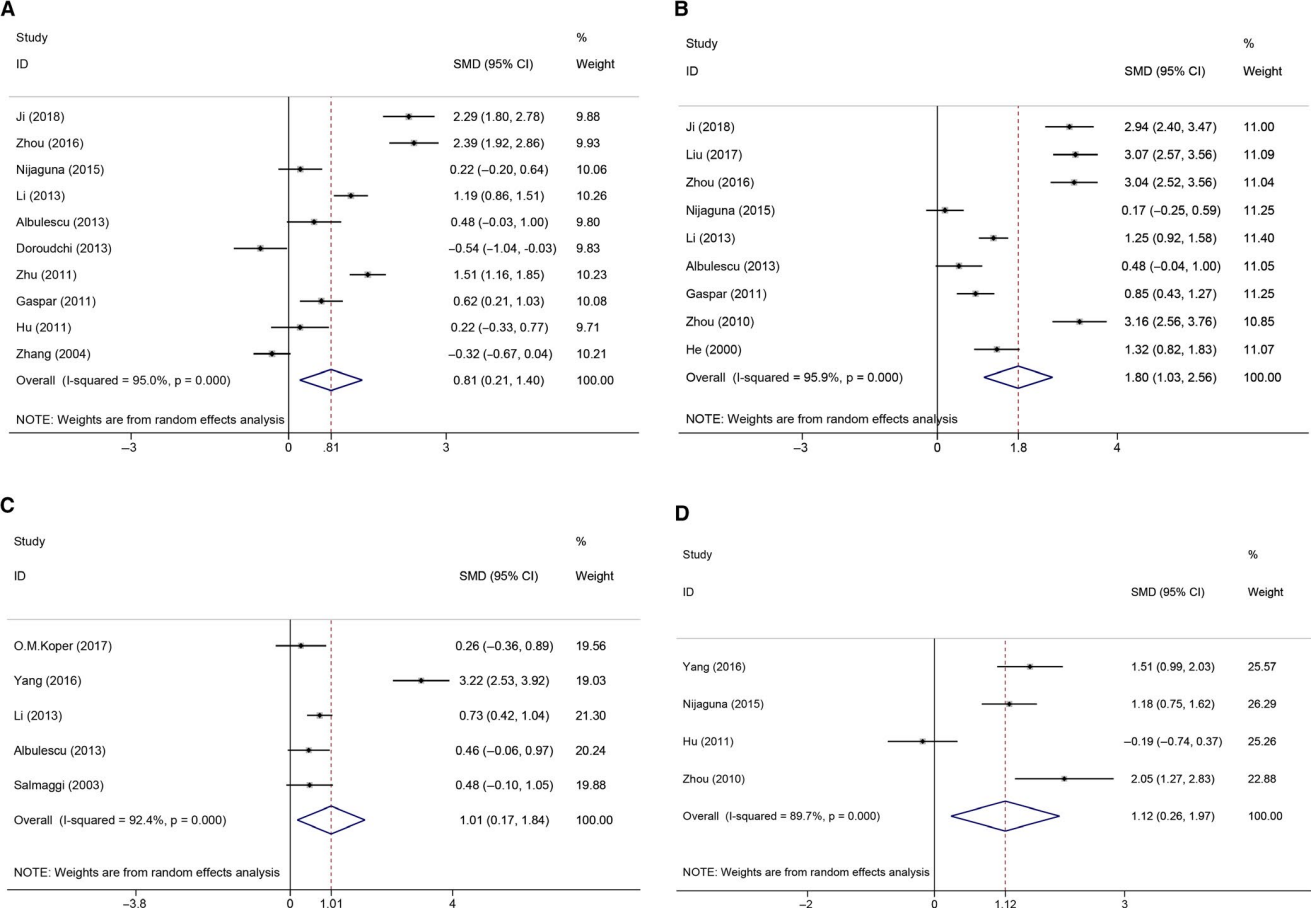
The forest plots of the association between circulating inflammatory factors and glioma risk. A, Association between IL‐6 and glioma risk. B, Association between TNF‐*α* and glioma risk. C, Association between IL‐8 and glioma risk. D, Association between IL‐17 and glioma risk

A total of 10 studies reported an association between IL‐6 and the pathogenesis of glioma. The pooled SMD was 0.81 (95% CI: 0.21‐1.40; *P* = .008), indicating that the level of IL‐6 (Figure 2A) in the peripheral blood of glioma patients was significantly higher than that of the normal control group. This result showed that glioma was associated with a higher IL‐6 level compared with the control. Because of the limited number of articles included in our study, it is not appropriate to conduct a meta‐regression analysis, so subgroup analyses are used to explore heterogeneous sources from regions, methods used for measurement, patient age, and tumor types. In the subgroup analyses of the samples described above (Table 3), the following predicted that elevated IL‐6 levels were associated with an increased glioma risk: region in Asia (pooled SMD 0.87 [95% CI: 0.13‐1.61; *P* = .021]); region in Europe (pooled SMD 0.57 [95% CI: 0.25‐0.89; *P* = .001]); using ELISA (pooled SMD 0.88 [95% CI: 0.02‐1.74; *P* = .044]); using other methods (pooled SMD 0.65 [95% CI: 0.01‐1.28; *P* = .046]); age ≥ 60 years (pooled SMD 0.57 [95% CI: 0.25‐0.89; *P* = .001]); age < 60 years (pooled SMD 0.96 [95% CI: 0.14‐1.79; *P* = .022]); GBM types (pooled SMD 0.44 [95% CI: 0.19‐0.70; *P* = .001]); and mixed gliomas (pooled SMD 1.18 [95% CI: 0.38‐1.98; *P* = .004]). Thus, it can be concluded that glioma secretes a large amount of IL‐6 into the peripheral circulation.

**Table 3:** Subgroup analyses of the association between circulating inflammatory cytokines and the glioma risk

Inflammatory factors	Subgroup analysis	Number of studies	Heterogeneity *I* ^2^ (%)	*P*_h_	SMD(95%CI)	*P‐*value
IL‐6	Region					
	Asia	8	96.0%	0.000	0.87 (0.13‐1.61)	.021
	Europe	2	0.0%	0.679	0.57 (0.25‐0.89)	.001
	Method					
	ELISA	7	96.4%	0.000	0.88 (0.02‐1.74)	.044
	Others	3	85.8%	0.001	0.65 (0.01‐1.28)	.046
	Age					
	≥60	2	0.0%	0.679	0.57 (0.25‐0.89)	.001
	<60	7	96.4%	0.000	0.96 (0.14‐1.79)	.022
	NA	1	/	/	0.22 (−0.20‐0.64)	.297
	Tumor types					
	GBM	3	0.0%	0.397	0.44 (0.19‐0.70)	.001
	Mixed	6	95.1%	0.000	1.18 (0.38‐1.98)	.004
	NA	1	/	/	−0.32 (−0.67‐0.04)	.079
IL‐8	Region					
	Asia	2	97.6%	0.000	1.96 (−0.49‐4.40)	.118
	Europe	3	0.0%	0.863	0.41 (0.08‐0.74)	.014
	Method					
	ELISA	3	95.8%	0.000	1.31 (−0.45‐3.08)	.144
	Others	2	0.0%	0.381	0.66 (0.39‐0.92)	.000
	Age					
	≥60	1	/	/	0.46 (−0.06‐0.97)	.084
	<60	3	95.8%	0.000	1.39 (−0.11‐2.88)	.069
	NA	1	/	/	0.48 (−0.10‐1.05)	.103
	Tumor types					
	GBM	1	/	/	0.46 (−0.06‐0.97)	.084
	HGG	1	/	/	0.48 (−0.10‐1.05)	.103
	Mixed	3	95.8%	0.000	1.39 (−0.11‐2.88)	.069
IL‐10	Region					
	Asia	2	0.0%	0.856	0.48 (0.24‐0.73)	.000
	Europe	3	96.4%	0.000	0.00 (−1.68‐1.68)	.999
	Method					
	ELISA	2	84.8%	0.010	0.79 (−0.20‐1.78)	.119
	Others	3	95.1%	0.000	−0.17 (−1.27‐0.92)	.755
	Age					
	≥60	1	/	/	−1.54 (−2.11– −0.97)	.000
	<60	3	78.4%	0.010	0.69 (0.13‐1.25)	.016
	NA	1	/	/	0.45 (0.03‐0.87)	.035
	Tumor types					
	GBM	3	96.5%	0.000	0.07 (−1.42‐1.56)	.927
	HGG	1	/	/	0.26 (−0.36‐0.88)	.407
	Mixed	1	/	/	0.50 (0.19‐0.81)	.001
TNF‐*α*	Region					
	Asia	7	96.2%	0.000	2.12 (1.22‐3.03)	.000
	Europe	2	15.5%	0.277	0.70 (0.34‐1.05)	.000
	Method					
	ELISA	4	95.6%	0.000	2.47 (1.29‐3.64)	.000
	Others	5	94.4%	0.000	1.26 (0.40‐2.12)	.004
	Age					
	≥60	2	15.5%	0.277	0.70 (0.34‐1.05)	.000
	<60	5	94.3%	0.000	2.68 (1.78‐3.58)	.000
	NA	2	91.6%	0.001	0.74 (−0.39‐1.87)	.201
	Tumor types					
	GBM	3	60.8%	0.078	0.50 (0.09‐0.92)	.018
	Mixed	4	95.1%	0.000	2.56 (1.52‐3.60)	.000
	NA	2	95.2%	0.000	2.23 (0.43‐4.03)	.015

Abbreviations: CI, confidence interval; ELISA, enzyme‐linked immunosorbent assay; NA, not available; GBM, glioblastoma multiforme; HGG, High‐grade glioma; SMD, Standard mean differences.

Additionally, glioma patients were significantly confirmed to possess higher circulating IL‐8 (Figure 2C) levels compared to controls, with a pooled SMD of 1.01 (95% CI: 0.17‐1.84; *P* = .018). In the subgroup analyses of regions (Table 3), we found that IL‐8 levels in the circulating blood of glioma patients were significantly increased in European populations, with a pooled SMD of 0.41 (95% CI: 0.08‐0.74; *P* = .014). However, Asian studies did not find a significant association between IL‐8 and glioma patients (pooled SMD 1.96 [95% CI: −0.49‐4.40; *P* = .118]). Based on the methodological subgroup analysis (Table 3), patients with glioma detected using other methods (pooled SMD 0.66 [95% CI: 0.39‐0.92; *P* = .000]) but not ELISA (pooled SMD 1.31 [95% CI: −0.45‐3.08; *P* = .144]) had significantly elevated levels of IL‐8. However, we did not find any association between circulating IL‐8 levels and glioma risk in the subgroup analyses of tumor types (Table 3).

**Figure 3:**
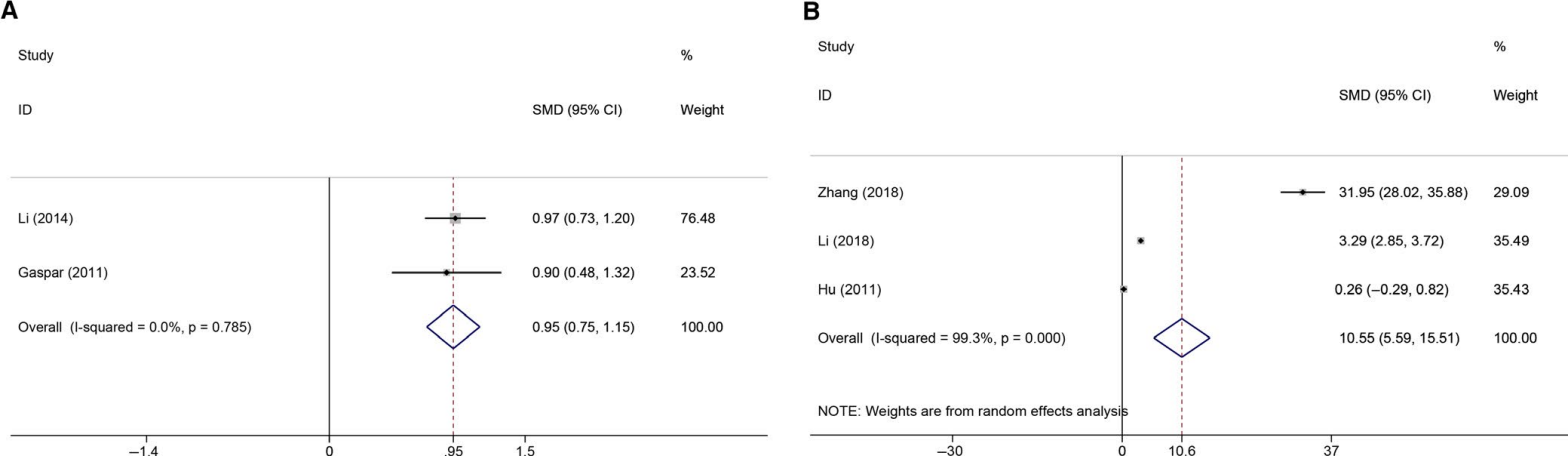
The forest plots of the association between circulating inflammatory factors and glioma risk. A, Association between CRP and glioma risk. B, Association between TGF‐*β* and glioma risk

Similarly, for TNF‐*α* (Figure 2B), the pooled SMD was 1.80 (95% CI: 1.03‐2.56; *P* = .000), indicating that an elevated level of TNF‐*α* in the peripheral blood is significantly associated with an increased glioma risk. In the subgroup analyses (Table 3), the pooled SMDs of research regions, measuring methods, patient age, and tumor types were 2.12 (95% CI: 1.22‐3.03; *P* = .000) for the Asian group and 0.70 (95% CI: 0.34‐1.05; *P* = .000) for the European group; 2.47 (95% CI: 1.29‐3.64; *P* = .000) for the ELISA group and 1.26 (95% CI: 0.40‐2.12; *P* = .004) for the other methods group; 2.68 (95% CI: 1.78‐3.58; *P* = .000) for age < 60 years and 0.70 (95% CI: 0.34‐1.05; *P* = .000) for age ≥ 60 years; and 0.50 (95% CI: 0.09‐0.92; *P* = .018) for GBM and 2.56 (95% CI: 1.52‐3.60; *P* = .000) for mixed gliomas, respectively. With respect to IL‐17 (Figure 2D), CRP (Figure 3A), and TGF‐*β* (Figure 3B), the pooled SMDs were 1.12 (95% CI: 0.26‐1.98; *P* = .011), 0.95 (95% CI: 0.75‐1.15; *P* = .000), and 10.55 (95% CI: 5.59‐15.51; *P* = .000), respectively.

**Figure 4:**
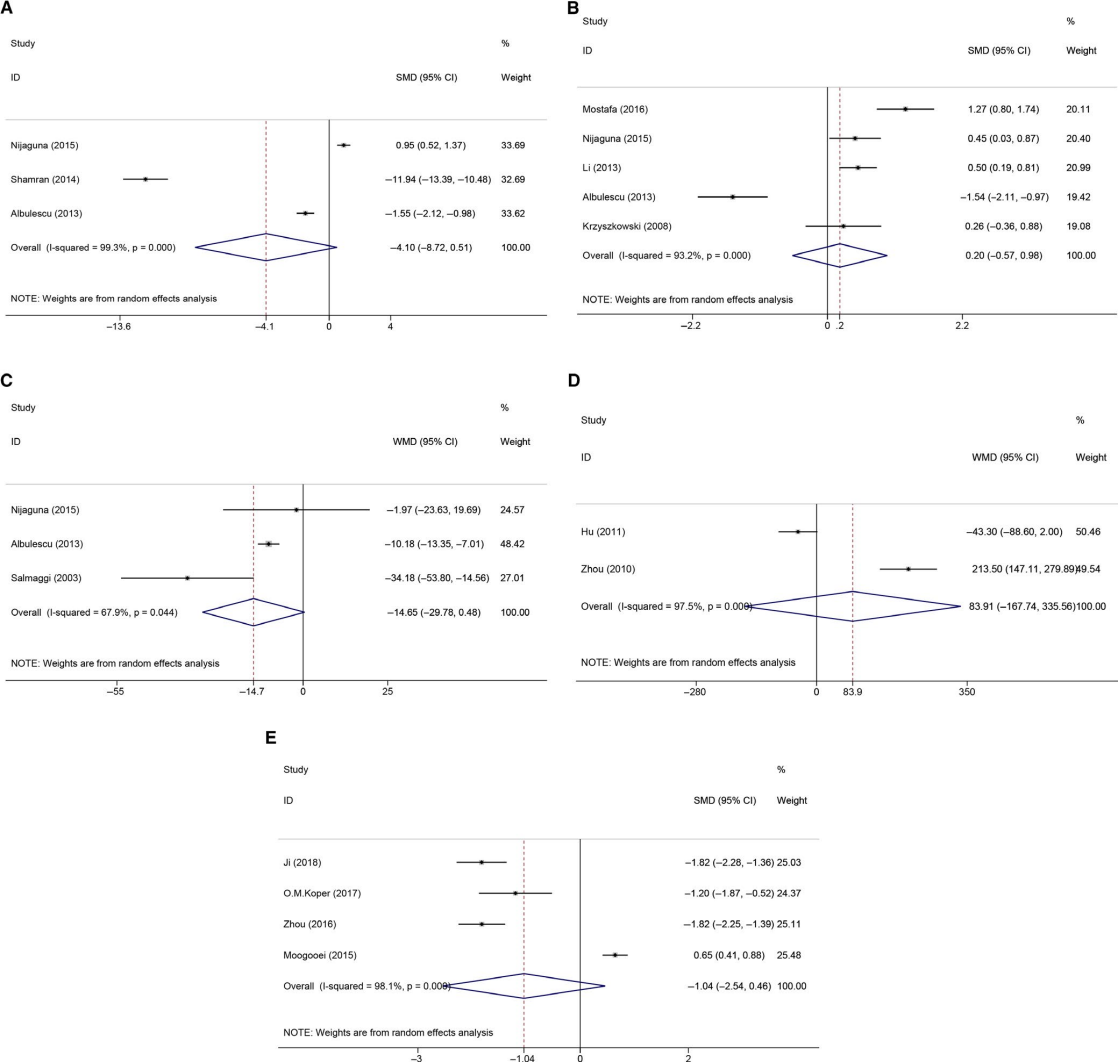
The forest plots of the association between circulating inflammatory factors and glioma risk. A, Association between IL‐4 and glioma risk. B, Association between IL‐10 and glioma risk. C, Association between IL‐12 and glioma risk. D, Association between IL‐23 and glioma risk. E, Association between MCP‐1 and glioma risk

We failed to find a significant association between circulating IL‐4 (Figure 4A), IL‐10 (Figure 4B), IL‐12 (Figure 4C), IL‐23 (Figure 4D), and MCP‐1 (Figure 4E) levels and glioma risk. The combined SMDs for IL‐4, IL‐10, and MCP‐1 were −4.10 (95% CI: −8.72‐0.51; *P* = .082), 0.20 (95% CI: −0.57‐0.98; *P* = .608), and −1.04 (95% CI: −2.54‐0.46; *P* = .175), respectively. For IL‐12 and IL‐23, we used WMDs to conduct the meta‐analysis. For these factors, the pooled WMDs were −14.65 (95% CI: −29.78‐0.48; *P* = .058) and 83.91 (95% CI: −167.74‐335.56; *P* = .513), respectively. However, when we performed the subgroup analyses based on regions and tumor types of the IL‐10 level (Table 3), we found that the circulating levels of IL‐10 in Asian patients (pooled SMD 0.48 [95% CI: 0.24‐0.73; *P* = .000]) and mixed gliomas (pooled SMD 0.50 [95% CI: 0.19‐0.81; *P* = .001]) were significantly higher than in healthy controls. Nevertheless, we did not find the same results in European studies (pooled SMD 0.00 [95% CI: −1.68‐1.68; *P* = .999]), GBM types (pooled SMD 0.07 [95% CI: −1.42‐1.56; *P* = .927]), and high‐grade glioma (HGG) types (pooled SMD 0.26 [95% CI: −0.36‐0.88; *P* = .407]). Additionally, in the subgroup analyses of ages for IL‐10 (Table 3), we found that increased circulating IL‐10 [SMD 0.69 (95% CI: 0.13‐1.25; *P* = .016)] was significantly associated with glioma risk for age < 60 years, and decreased circulating IL‐10 [SMD −1.54 (95% CI: −2.11 to −0.97; *P* = .000)] was significantly associated with glioma risk for age ≥ 60 years.

Distinct heterogeneity was observed in studies investigating the inflammatory factors mentioned above (IL‐4, IL‐6, IL‐8, IL‐10, IL‐12, IL‐17, IL‐23, TNF‐*α*, TGF‐*β*, and MCP‐1), with *I* ^2^ above 50%. Considering the subgroup analyses of IL‐6, heterogeneity was reduced in European studies (*I* ^2^ = 0.0%, *P*_h_ = 0.679), studies that examined patients aged ≥ 60 years (*I* ^2^ = 0.0%, *P*_h_ = 0.679), and GBM studies (*I* ^2^ = 0.0%, *P*_h_ = 0.397). With regard to IL‐8, heterogeneity was reduced in the subgroups of European studies (*I* ^2^ = 0.0%, *P*_h_ = 0.863) and studies using other methods (*I* ^2^ = 0.0%, *P*_h_ = 0.381). For TNF‐*α*, heterogeneity was reduced in European studies (*I* ^2^ = 15.5%, *P*_h_ = 0.277) and in those that examined patients aged ≥ 60 years (*I* ^2^ = 15.5%, *P*_h_ = 0.277). Moreover, heterogeneity was reduced in Asian studies (*I* ^2^ = 0.0%, *P*_h_ = 0.856) about IL‐10. Thus, we can speculate that heterogeneity may be attributed to the research region, the mean age of glioma patients, and the tumor types.

### 3.3 Prognostic significance of circulating inflammatory factors in glioma patients

**Figure 5:**
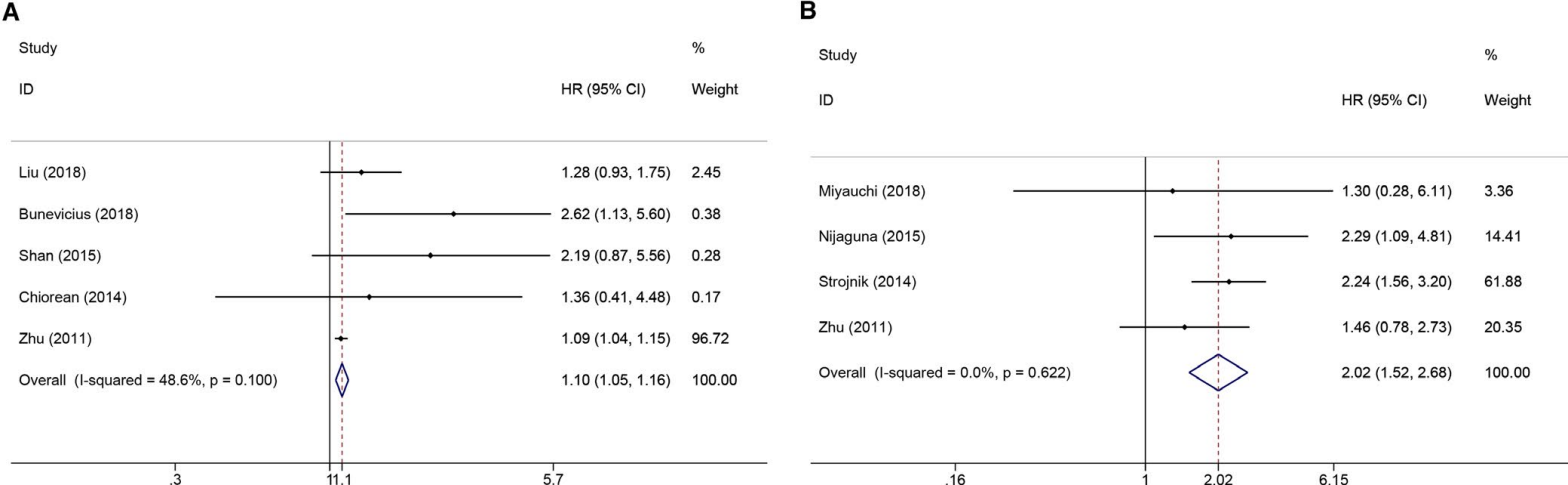
The forest plots of the prognostic significance of circulating inflammatory factors in glioma patients. A, The prognostic value of IL‐6 in glioma patients. B, The prognostic value of CRP in glioma patients

Five studies with 535 glioma patients were included in the prognostic analysis for IL‐6 (Figure 5A). The pooled HR was 1.10 (95% CI: 1.05‐1.16; *P* = .000), indicating that an elevated circulating IL‐6 level predicted poor OS in glioma patients. There was no conspicuous heterogeneity between the included studies (*I* ^2^ = 48.6%, *P*_h_ = 0.100). Regarding CRP (Figure 5B), four studies including 466 glioma patients were selected. The expression of circulating CRP levels was significantly correlated with poor OS in glioma patients, and the pooled HR was 2.02 (95% CI: 1.52‐2.68; *P* = .000). Additionally, no significant heterogeneity was found between the included studies (*I* ^2^ = 0.0%, *P*_h_ = 0.622). These results provide more credible evidence for the prognostic significance of circulating IL‐6 and CRP levels in glioma patients.

## 4 SENSITIVITY ANALYSIS AND PUBLICATION BIAS

**Figure 6:**
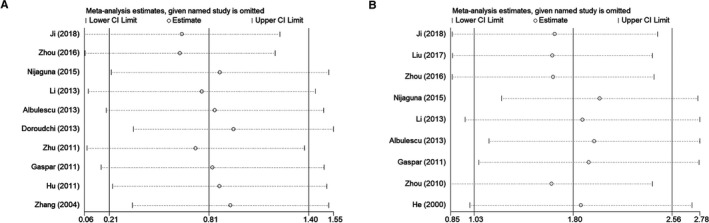
Sensitivity analysis of the association between circulating inflammatory factors and glioma risk. A, Sensitivity analysis of the association between IL‐6 and glioma risk. B, Sensitivity analysis of the association between TNF‐*α* and glioma risk

**Figure 7:**
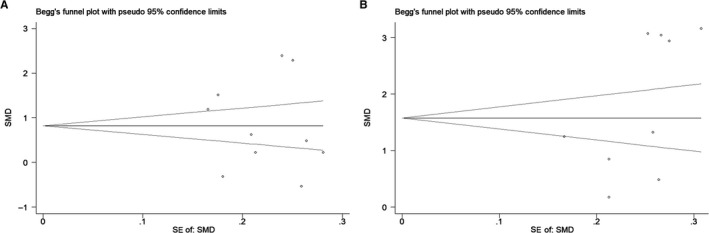
Begg's test results of the association between circulating inflammatory factors and glioma risk. A, Begg's test result of IL‐6 and glioma risk. B, Begg's test result of TNF‐*α* and glioma risk

We conducted a sensitivity analysis and examined publication bias of the meta‐analysis on the relationship between IL‐6 (Figure 6A) and TNF‐*α* (Figure 6B) expression levels and glioma risk. No individual study markedly affected the pooled SMD based on the sensitivity analysis, which indicates that the SMD estimates are stable and reliable. Publication bias was assessed using Begg's and Egger's tests. For IL‐6, Begg's test (*z* = 0.36, *P*>|*z*| = .721) revealed no evidence of publication bias among the 10 studies (Figure 7A), and Egger's test (*t* = −0.14, *P*>|*t*| = .891) showed the same conclusion. Regarding TNF‐*α*, Begg's test (*z* = 1.15, *P*>|*z*| = .251) revealed no evidence of potential publication bias among the nine studies (Figure 7B), and Egger's test (*t* = 1.80, *P*>|*t*| = .115) also showed no bias.

## 5 DISCUSSION

In tumor areas, inflammatory cytokines and their receptors form a comprehensive regulatory network, which plays a significant role in the development and progression of cancer. Currently, inflammatory factors are highlighted in cancer diagnosis, prognosis, and therapy. However, the research conclusions of biomarkers related to gliomas remain unclear. The present meta‐analysis summarized the evidence to demonstrate the relationship between circulating inflammatory factors and the risk of glioma as well as their prognostic values in glioma from 31 included articles. The results showed that increased circulating IL‐6, IL‐8, IL‐17, TNF‐*α*, TGF‐*β*, and CRP levels before treatment are significantly associated with glioma risk. Furthermore, no significant association was found between preoperative circulating IL‐4, IL‐10, IL‐12, IL‐23, and MCP‐1 levels and glioma risk. On the other hand, our results showed a poor prognostic outcome in glioma patients expressing high levels of circulating IL‐6 and CRP.

In the tumor environment, inflammatory factors are responsible for cell proliferation, tumor invasion, significant angiogenesis, and the suppression of certain immune functions to promote the occurrence and development of tumors.^46^ The tight junction between endothelial cells is the main structural and functional basis of the blood‐brain barrier.^47^ Normal brain capillary endothelial cells have no window structure.^47^ Some studies have shown that there are obvious changes in the morphology of capillaries forming the blood‐brain barrier in glioma.^48^, ^49^, ^50^ The appearance of the window structure, and the appearance of wormlike holes in the basement membrane may explain the changes in inflammatory factors in the blood. Albulescu et al found altered serum profiles of inflammatory factors (such as IL‐6, IL‐8, and TNF‐*α*) in glioma patients that were closely linked to brain tumor behavior.^37^ IL‐6 is a multifunctional cytokine that can induce the proliferation and differentiation of immune cells. The tumor cells themselves and immune cells surrounding the tumor can release IL‐6, thereby increasing circulating IL‐6 levels. Elevated levels of serum IL‐6 associated with tumor size, tumor stage, or disease progression have been found in many neoplastic diseases, such as gastrointestinal tumors, lung cancer, and malignant melanoma.^51^ Trikha et al revealed that the serum IL‐6 level correlated with clinical extension of the tumor and with outcomes.^52^ In our study, we found that an elevated circulating IL‐6 level is significantly associated with an increased risk and a poor prognosis of glioma, probably because it is involved in the occurrence and development of glioma. Much evidence suggests that IL‐6 plays a significant role in the development of glioma by promoting angiogenesis and cell proliferation and because of its antiapoptosis and antiradiation effects.^9^, ^53^, ^54^ In addition, studies have shown that the serum IL‐6 level is positively correlated with the expression of IL‐6 protein in glioma tissues, as detected by immunohistochemistry, suggesting that IL‐6 may participate in the progression of glioma in an autocrine or paracrine manner.^53^, ^55^ Based on the evidence described above, it is easy to understand that an elevated IL‐6 level is closely associated with the risk and prognosis of glioma patients.

CRP is a classical nonspecific acute‐phase protein produced in the liver.^34^ Epidemiological studies have suggested that for several types of solid cancer, elevated circulating levels of CRP are associated with poor outcomes.^13^ Based on the particular relationship between tumors and inflammation, a sharp increase in CRP content in the presence of inflammation makes it a reliable biomarker to reflect the relationship between inflammation and cancer. The results from our present meta‐analysis showed a high risk of developing glioma with elevated levels of CRP and a poor prognostic outcome in glioma patients expressing high levels of CRP. Possible mechanisms for high CRP levels associated with an increased risk and a poor prognosis of glioma have been reported.^12^ On the one hand, CRP protects endothelial cells from starvation‐induced death by acting on microglia through IL‐1*β*, which contributes to tumor angiogenesis and progression. On the other hand, IL‐6 secreted by GBM acts on hepatocytes and may secrete high levels of CRP through the janus kinase‐signal transducer and activator of transcription (JAK‐STAT) pathway, which reaches the tumor site through blood circulation and then accumulates in tumor tissue. These phenomena also suggest that our conclusions about IL‐6 are credible and accurate.

Moreover, we found that increased circulating IL‐8, IL‐17, TNF‐*α*, and TGF‐*β* levels are significantly associated with glioma risk, which indicates that these inflammatory factors are involved in the pathogenesis of glioma. IL‐8 is a potent angiogenic factor for the progression of malignant gliomas and is correlated with the histopathological grade of gliomas.^56^ Studies have indicated that GBM cells, which secrete IL‐8, promote angiogenesis and microvascular endothelial permeability.^57^ Over the past few years, the possible role of IL‐17 in tumors has received increasing attention. The function of IL‐17 in tumors may include the promotion of angiogenesis by upregulating vascular endothelial growth factor (VEGF) and CD31^58^, and/or the promotion of tumorigenesis through the IL‐6‐STAT3 signaling pathway.^59^ Hu et al reported that IL‐17 may influence glioma tumorigenesis and progression through certain signaling pathways.^39^ Through in vivo and in vitro studies, IL‐17 was shown to induce the proliferation and migration of glioma cells by activating PI3K/Akt1/NF‐κB‐p65.^60^ Accumulating evidence has shown that the pleiotropic functions of TNF‐*α* range from antitumor activity to tumorigenesis. The characteristic of TNF‐*α* inhibition is that it binds to malignant tumor cells, changes gene expression in cells, destroys the cell cycle, and then causes the tumor cells to no longer proliferate indefinitely.^61^ TNF‐*α* secretion leads to the promotion of glioma formation and development through angiogenesis.^62^ The expression of TGF‐*β* can be elevated in a variety of malignant tumors and is closely related to the invasive growth, metastasis, and other processes of the tumor.^63^ In particular, TGF‐*β* was shown to correlate with the poor prognosis of advanced stages of glioma.^64^ In conclusion, circulating IL‐6, IL‐8, IL‐17, TNF‐*α*, TGF‐*β*, and CRP levels may be reliable biomarkers describing the risk of glioma. Furthermore, the circulating IL‐6 or CRP level may be a reliable prognostic indicator in glioma patients.

Our results showed no significant association between circulating IL‐4, IL‐10, IL‐12, IL‐23, and MCP‐1 levels and the risk of glioma patients. The primary reason for this finding may be that the number of studies included was limited, and the sample size of each study was not large. On the other hand, the negative conclusions are closely related to the various characteristics of these inflammatory factors involved in tumor progression. Therefore, further studies with large sample sizes are necessary to establish the true correlation as well as the unique pathogenesis.

In this meta‐analysis, there are unavoidable limitations in several aspects that should be further considered. First, the regions and populations involved in our study were limited (only Asia and Europe). Second, the cut‐off values for inflammatory factors were different between the included studies, which could increase heterogeneity among studies. Third, because of the lack of studies relating the expression of other inflammatory factors and OS (except for IL‐6 and CRP), we could not draw an exact conclusion. Fourth, based on the incomplete data about different pathological types of gliomas in original studies, we did only a few subgroup analyses of glioma risk in different pathological types, and cannot draw a complete conclusion. Finally, although we conducted subgroup analyses for IL‐6, IL‐8, IL‐10, and TNF‐*α* with glioma risk to explore the potential sources of heterogeneity, high heterogeneity still existed in the studies of IL‐4, IL‐12, IL‐17, IL‐23, TGF‐*β*, and MCP‐1 based on the limited number of included studies.

## 6 CONCLUSIONS

In conclusion, our meta‐analysis suggested that circulating IL‐6 and CRP levels may serve as powerful biomarkers for a poor prognosis in glioma patients. Determination of the levels of circulating IL‐6 and CRP might aid in predicting the clinical outcome in glioma patients. Moreover, our results indicated that increased circulating IL‐6, IL‐8, IL‐17, TNF‐*α*, TGF‐*β*, and CRP levels are significantly associated with increased glioma risk. Additionally, based on the results of subgroup analyses of glioma risk in different pathological types, we could better understand the various characteristics of inflammatory factors involved in tumor progression. To investigate this problem deeply, studies with large sample sizes, covering extensive areas, and with complete data about different pathological types of gliomas are required. Considering that the acquisition and measurement methods of blood samples are relatively easy, we believe that the detection of circulating inflammatory factors and an in‐depth study of their mechanisms of promoting tumorigenesis could provide a broader prospect for reducing glioma risk and supplementing methods of predicting the outcome in glioma patients.
